# Bio-objects and the bio-objectification process

**DOI:** 10.3325/cmj.2011.52.740

**Published:** 2011-12

**Authors:** Tora Holmberg, Nete Schwennesen, Andrew Webster

**Affiliations:** 1 Institute for Housing and Urban Research, Gävle, Sweden *tora.holmberg@ibf.uu.se*; 2 Steno Diabetes Center, Gentofte, Denmark *nesc@sund.ku.dk*; 3 SATSU, University of York, UK *andrew.webster@york.ac.uk*

This short text is the second in a series of articles from the recently established “Bio-Objects” research network supported by the European Commission’s Cooperation in Science and Technology (COST) program (1). Here we explore in more detail the ways in which we understand the boundaries of bio-objects determined through a “bio-objectification” process wherein life-forms or living entities are first made into objects, become possible, through scientific labor and its associated technologies, and then come to be attributed with specific identities. This move from living entity, through bio-objectification to what we can call “bio-identification” helps us to understand the contested, often controversial process seen in the biological sciences (and not merely in biomedicine, but elsewhere, such as in agriculture and food research) where we see a new mixture of relations to life or to which “life” is attributed, such as animal-human hybrids, chimera, genetically modified organisms, or transgenics. As a consequence of these novel relations, the boundaries between human and animal, organic and nonorganic, living and the suspension of living (and the meaning of death itself), are often questioned and destabilized, and their identities have to be negotiated and (temporarily) stabilized, and so given an identity. What is common to what we call bio-objects, is that they all in various ways challenge conventional cultural, scientific, and institutional orderings and classifications.

One obvious boundary to address is the one between humans and other animals. Bio-medical innovations such as transgenic and cloned animals, or hybrid embryos, transgress species boundaries that we like to think of as given by nature. These novel bio-objects potentially point out that the natural order is rather a social one, or perhaps better, a bio-social one. However, this potentiality is not often realized. These objects, when traveling through various regulatory bodies in society, including the bio-ethical sphere, are treated as special cases that rather confirm, than move, species boundaries. For example, from the European Chimbrids project, which dealt with the ethical and legal regulations of various forms of species mixes, it becomes clear that these mixes re-instate the discourse of human superiority, as markers of “human-ness” are constantly being emphasized (for example brain size, language, rationality, and genomic distinctions).

A second example that is of interest is the living/non-living boundary. Novel bio-objects often challenge or transgress what we conventionally refer to as a clear line between living and non-living. For example when it comes to human fetuses in research and bio-medical practice – whether it concerns embryonic stem cells or genetic preimplantation testing in in-vitro fertilization, the boundaries between what is considered a living organism are negotiated in clinical practice, laboratories, media, and regulatory bodies. The boundaries also shift depending on the context, thus implying that we are dealing with different bio-objects. In Sweden, the limit is set at 14 days for embryonic research, and 18 weeks for abortion. While the first limit has been debated lately in relation to stem cell research, the latter is not open for debate. The arbitrariness of living/non-living boundaries points at many interesting and highly political openings.

These examples of dynamic processes suggest that there is no once-and-for-all list of bio-objects, a sort of bio-object catalog or portfolio, made up of life forms that have specific properties or essential characteristics that make them inherently bio-objects. Instead, we have to begin from the position that bio-objects are created in and through developments in science that leads to debate over the meaning of life and that make life matter. It need not, therefore, be the novel materiality of life that itself kick-starts the bio-objectifying process.

From a research point of view, this suggests we need to identify different arenas or locations where bio-objectification is to be found – eg, in controversial clinical trials (such as the Geron stem cells trial), contested diagnostics (typically within the field of genomics or imaging), or the domain of reproduction (such as in debates over the boundaries between viable and non-viable life) – and how the boundaries of life and emergent bio-identities are enacted within these arenas. This also raises the more challenging question of whether traffic between different sites translates and transforms the bio-objectification process. A bio-object, associated with, say, biomedical research, may subsequently find its way into the food system or the environment, or become part of a repository and new medium of technical innovation (to be stored in biobanks or cord blood banks), and have multiple or even contrasting cultural meanings as it circulates between different sectors or networks of society. At the same time, new regulatory boundaries are developed for which human and non-human material can and cannot be legitimately traded as bio-objects (for example, oocytes and embryos).

It should be clear from what we have said that the concept of bio-objectification is much richer than concepts found elsewhere in the social science literature such as the “molecularization” of life, which claims that biological life is now understood through molecular biology and genetics. Rather, we seek to explore the articulation of this process, and other science-based constructions of life. It should be stressed that bio-objectification processes are not linear or have a specific path-dependency. Bio-objectification can start at one point, go through institutional transformations, come to a halt or be silenced, and then revitalized at a later point. This means that bio-objectification explicitly includes consideration of organizational and institutional processes and the ways in which the governance of bio-objects can bring closure and stability to them, but which is always likely to leave open the possibility of new contestation and debate in the future, and, of course, across different countries, even at the same time.

We argued above that bio-objects are in principle, contested socio-technical objects. But they depend on the existence and manipulation of living entities that have some coherent biological form and agency. This does not mean that the biological entities are “naked” or completely plastic before they become subject to the bio-objectification process. There are some biomaterial “affordances” to the entities that limit their variability and use and forms and degrees of manipulation and control exercised over them by bioscience itself. Science seeks to stabilize and classify and deploy bio-objects in novel ways, but this can be extremely difficult, as we have seen in recent years in attempts to standardize and control the use of embryonic stem cells in cell therapies.

As manipulated bio-objects appear, precisely because they are characterized as constructed, as having a hybridity involving the manipulation and capture of biological forms and processes, with no “intrinsic” self or boundary as such, they often evoke the language of the “unnatural” or, in some way, being disconnected from “the normal.” This reminds us of the work of the social anthropologist Mary Douglas, who wrote about “matter out of place” – cultural and material forms that do not fit into existing taxonomies, threaten order, and because of this, need to be brought under some sort of control. They are thus dealt with through various neutralizing strategies. At the same time, not often acknowledged in Douglas’ work, these unruly objects are ambiguous in their effects, they do not only constitute threats, but can also provide a positive force. Consequently, such “monsters” need to be reined in through regulatory “heroes,” whose task is to tame the unruly object, or at least to provide stabilizing mechanisms – within the political arena these might be various forms of deliberative debate/consensus conferences and regulations – that establish the terms on which engagement with the hybrid monster – the genetically modified organism, the xenotransplant etc – is to be undertaken. This process also helps to provide a framework through which markets for bio-objects become possible, and indeed promoted, as we see in the expectations (often overstated) surrounding the growth of regenerative medicine. In addition, we see a range of scientific, regulatory, and legal provisions and conventions appear, such as the determination of species boundaries, the ethical rights and obligations of the bio-object and those using it, and diverse provisions associated with claims to ownership of property (including property of a body).

Bio-objects are then about material life that changes the meaning of life and gives it new trajectories through the resolution process associated with bio-objectification and thereafter bio-identification. But we can identify another form of “vitality” that has agency that is not fully encompassed by this concept, yet which is often important in defining and making mobile (both geographically and scientifically across different areas of inquiry) bio-objects themselves. We can call this the domain of the “bio-virtual,” a specific form of life which exists as information, data and informational flows that mobilizes bio-objects through data networks as a form of aggregative life. These different forms of vitality are summarized in Table 1.

**Table 1:** Different forms of vitality

	Living entity	Bio-object	Bio-virtual
**Socio-material identity**	Viable biological material	Hybridized/manipulated biological life	Aggregative life
**Example**	Human or animal	Human embryonic stem cell	Biodata

**Figure 1:**
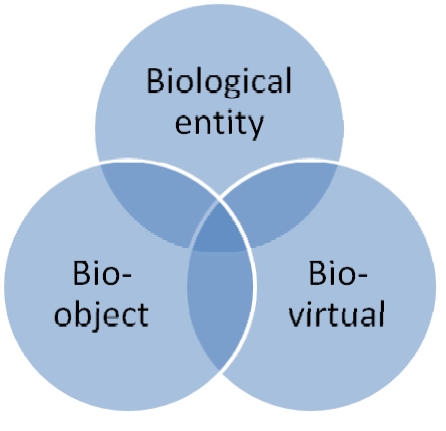
Three related dimensions of biological entities, bio-objects, and the bio-virtual

Moreover, we outline below how our three related dimensions of biological entities, bio-objects, and the bio-virtual come together (Figure 1). The substantive and methodological focus for our work in the COST Action will be on the ways these relate as in the diagram below, crucially in the processes of bio-objectification and bio-identification found in the center of the diagram.

The main methodological implications of the argument thus far are 3-fold – we need to engage in:

• Mapping and tracking: follow the bio-object as it engages (circulates across) current legal provisions and regulation – understanding the bio-objectification process and its different dimensions

• Comparing: determining the divergences and convergences of bio-objects/objectification across different domains

• Modeling: identifying stabilizing (shape-holding) and disruptive patterns and processes associated with bio-objectification

The COST Action will draw on members’ substantive research interests to undertake these three overlapping tasks.

To summarize, the concept of bio-object stresses the point that boundaries around “the living” are not stable and that there is what we may call a potential openness in processes of bio-objectification and bio-identification through which such boundaries are drawn. In other words, it is not given what will count as categories of life, such as human or animal, viable life or non viable life, biological or social. Where the boundaries get drawn and what meaning categories get assigned to, are crucial in terms of knowledge production, bio-political interventions and regulations, and everyday lives in a more-than-human world. When, where, how, and with what results such boundaries are made and negotiated, are interesting and politically charged questions to ask.
